# Comparison of treatment efficacy of omega-3 fish oil and montelukast in ovalbumin-protease-induced allergic rhinitis model in rats

**DOI:** 10.1016/j.bjorl.2024.101399

**Published:** 2024-02-21

**Authors:** Alper Tabaru, Sahin Ogreden, Salih Akyel, Mehmet Faruk Oktay, Kemal Uslu, Funda Kaya Emre

**Affiliations:** a University of Health Sciences, Basaksehir Cam ve Sakura City Hospital, Otolaryngology Department, Istanbul, Turkey; b University of Health Sciences, Bagcılar Training and Research Hospital, Otolaryngology Department, Istanbul, Turkey; c University of Health Sciences, Bagcılar Training and Research Hospital, Pathology Department, Istanbul, Turkey

**Keywords:** Allergic rhinitis, Fish oil, Omega-3, Montelukast, Nasal symptoms

## Abstract

**Objectives:**

Montelukast is a well-known leukotriene receptor antagonist commonly used in treating allergic rhinitis and asthma. Omega-3 fatty acid is also known as an antiallergic and immunomodulator molecule. This study aimed to elucidate the efficacy of systemic montelukast and omega-3 fatty acid treatment in allergic rhinitis models in Wistar Hannover rats.

**Methods:**

This research was conducted on 28 healthy Wistar Hannover rats weighing 250–350 g. After establishing the allergic rhinitis model, nasal symptoms were observed and scored, and the nasal mucosa of all rats was investigated histologically. Light microscopy was utilized to evaluate the degree of ciliary loss, goblet cell hyperplasia, vascular congestion, vascular proliferation, inflammatory cell infiltration, eosinophil infiltration, and hypertrophy in chondrocytes.

**Results:**

As a result of the analysis of the data obtained from the study, it was determined that typical allergic rhinitis symptoms such as nasal scratching and sneezing were significantly reduced in the rats in the montelukast and omega-3 treated group, and these symptoms did not increase after repeated intranasal OVA-protease applications. Histological examinations after fish oil treatment did not reveal typical inflammatory changes in allergic rhinitis. None of the rats in the montelukast and omega-3 groups had any increase in goblet cells, whereas 14.3% of the rats in the control group and 28.6% of the rats in the allergic rhinitis group had mild increase. Last but not least, 71.4% of rats in the allergic rhinitis group had a moderate increase. The difference between the groups was statistically significant (*p* < 0.001).

**Conclusion:**

Regarding the outcomes of this research, it was observed that w-3 fatty acids had antiallergic effects, both histopathological and clinical, in the allergic rhinitis model. We believe that further randomized controlled trials incorporating larger cohorts are warranted to verify the use of omega-3 fatty acids in treating allergic rhinitis.

The level of evidence of this article is Level 2.

## Highlights

• Omega-3 fatty acid is known as an antiallergic and immunomodulator molecule.
• Allergic rhinitis models can be created on experimental animals.
• The efficacy of fish oil in the allergic rhinitis model can be evaluated.

## Introduction

Rhinitis is an inflammation of the nasal mucosa. It is a clinical condition characterized by two or more symptoms, such as runny nose, sneezing, nasal congestion, and itching, usually for over one hour in two or more consecutive days. Rhinitis is a common morbidity that not only decreases the quality of life but also has severe effects on job and school performance, leading to a significant increase in healthcare expenditures.^1^ According to the Allergic Rhinitis and its Impact on Asthma (ARIA) guidelines, rhinitis is classified into two distinct groups: Allergic Rhinitis (AR) and Non-Allergic Rhinitis (NAR), based on etiology.

AR is an inflammatory disease of the nasal mucosa caused by immunoglobulin Ig E after contact with allergens, leading to symptoms such as runny nose, itching, sneezing, and congestion. AR is estimated to affect about 20%–40% of the world's population, and its prevalence has increased in recent years.^2^ Due to the symptoms of AR, serious distractions, adverse effects on cognitive functions, snoring, sleep disorders, restrictions, and failures in school and work performances are observed in affected people. Furthermore, significant psychological disturbances and adverse effects on social life are also observed in individuals with AR. Medications used in the treatment of AR can be listed as topical and oral antihistamines, topical and oral corticosteroids, topical and oral decongestants, mast cell stabilizers, mucolytics, anticholinergic agents, and nasal irrigation solutions.^3^

Essential fatty acids are present in various body parts, including cell membranes. They affect the viscosity of cell membranes, change their composition, and play an essential role in immune reactions by modulating cell signaling.^4^ One factor that plays an essential role in developing allergic rhinitis is nutrition. In the SAAC Phase I study, rice grain consumption was shown to be inversely related to the AR prevalence.^5^ The Mediterranean diet, which includes the consumption of grain, fish oil, fish, and other seafood, has been reported to generally reduce the risk of AR.^6^

Within the scope of this research, we aimed to elucidate the therapeutic efficacy of omega-3 fatty acids in preventing allergic rhinitis symptoms in Wistar Hannover rats.

## Methods

All procedures followed ethical standards set by the responsible committee on animal experimentation (institutional and national). Our institution (Bagcilar Training and Research Hospital) has granted ethics committee approval with protocol number 2016/145. This research was conducted on 28 healthy, 2–4 months old Wistar Hannover rats weighing 250–350 g. The rats were divided into four groups: Group 1 (control group), Group 2 (allergic rhinitis model without any treatment), Group 3 (allergic rhinitis model treated with montelukast), and Group 4 (allergic rhinitis model treated with omega-3 fatty acid). All animals were kept at 22 °C room temperature for 12 -hs in a dark/light cycle and fed with standard food daily (Table 1).

**Table 1:** Description and Number of animals in groups.

Experimental Groups	Number of animals
Group 1 Control Group	7
Group 2 Allergic Rhinitis Control Group	7
Group 3 AR Group given montelukast treatment	7
Group 4 AR Group given omega-3 fatty acid oil	7

AR, Allergic Rhinitis.

The study drugs were prepared via weighing on the Sartorius precision scale (GD603-0CE Carat Scale, Sartorius Mechatronics, Goettingen, Germany). Antigen solution was prepared as 0.3 mg Ovalbumin (OVA, Grade V, Sigma-Aldrich Chemical Co. St. Louis, MO) in 1 mL 0.9% saline and 30 mg aluminum hydroxide intraperitoneally (second, third, and fourth groups). Sensitization was created by applying once every two days for 14 days between 11:00–12:00. In the control group, the animals were intraperitoneally administered 1 mL of 0.9% saline intraperitoneally, once every two days for 14-days between 11:00‒12:00.

In the second stage of the study, to create an AR model in the sensitized animals, 1.0 mg/mL OVA in 0.9% SF and 0.54 U protease from Aspergillus oryzae was centrifuged, and 30 μL of the solution was instilled into both nostrils with a micropipette every day for 15 days. Alongside to the OVA + protease application, the third group received a 10 µL montelukast solution one hour before OVA application for 15 days via gavage. The rats in the fourth group were fed a mixture containing 8% omega-3 fatty acid. Ten microL 0.9% saline was applied to both nostrils of the animals in the control group for 14 days. Omega-3 and montelukast were initialized from the fifteenth study day and continued daily. Twenty-four hours after the drug application, all animals were sacrificed.

Subjective evaluation of allergic rhinitis symptoms was performed on days 1, 14, 17, 20, 23, 26, and 28 following intranasal OVA application after a 10-minute adaptation period with one animal in each cage. Nose scratching and sneezing were evaluated by the same physician.

### Statistical analysis

Average, standard deviation and median values were used in the descriptive statistics of the data. The distribution of variables was measured with the Kolmogorov-Smirnov Test. Cross-table (Chi-Square) analysis was used to analyze non-numerical variables. The Kruskal Wallis H test was utilized to analyze numerical variables, and the Mann-Whitney *U* test was performed to determine the difference between groups. Sneezing and nasal scratch count values were analyzed via the Mann-Whitney *U* test. Wilcoxon Ordered Numbers Test was conducted for group comparisons. A *p*-value of < 0.05 was considered statistically significant.

## Results

Following the first topical intranasal OVA + protease application, typical allergic rhinitis symptoms such as sneezing, nasal scratching, and runny nose were observed in the second, third, and fourth groups. These symptoms persisted for an average of 1.5 h.

The number of sneezes on the 14, 17, 20, 23, 26, and 28 days in group 2 was significantly higher than on day 1 (*p* = 0.019, *p* =  0.019, *p* = 0.019, *p* = 0.017, *p* = 0.017, *p* = 0.018). In the group treated with montelukast, there was a significant decrease in the number of sneezes since the third day of treatment. The sneezing was significantly higher in group 3 on days 14 and 17 (*p* = 0.017, *p* = 0.018, respectively), but no statistical significance has been observed on days 20, 23, 26, and 28.

The number of sneezes on days 14 and 17 was significantly higher in the fourth group compared to day 1 (*p* = 0.012), while the significance disappeared on days 20, 23, 26, and 28 (*p* = 0.26) (Table 2).

**Table 2:** Average number of sneezes by groups.

Groups	Day 1	Day 14	Day 17	Day 20	Day 23	Day 26	Day 28
Group 1 Control Group	1.27	1.14	1.60	1.56	1.84	2.00	2.00
Group 2 Allergic Rhinitis Control Group	1.27	8.68^a^	14.28^a^	17.42^a^	20.43^a^	21.20^a^	22.80^a^
Group 3 AR Group given montelukast treatment	1.14	8.56^a^	5.43^a^	1.84^b^	1.56^b^	2.00^b^	1.70^b^
Group 4 AR Group given omega-3 fatty acid oil	1.14	8.57^a^	5.45^a^	1.86^b^	1.57^b^	2.00^b^	1.74^b^

AR, Allergic Rhinitis.

a *p* <  0.05.

b *p* >  0.05.

In the second group, the number of nose-scratching movements on the 14th, 17th, 20th, 23rd, 26th, and 28th days significantly increased compared to day 1 (*p* = 0.018, *p* = 0.018, *p* =  0.016, *p* = 0.018, *p* =  0.016, *p* = 0.015, respectively). In the third group treated with montelukast, a significant decrease was observed in the number of scratching movements from the third day of treatment. The third group determined that the number of scratching movements on the 14th and 17th days was significantly higher than on the first day (*p* = 0.017, *p* =  0.018, respectively). On the other hand, the number of scratching movements on the 20th, 23rd, 26th, and 28th days was not significantly different from the first day (*p* = 0.078, *p* = 0.271, *p* = 0.089, *p* = 0.298, respectively).

The sneezing was significantly higher in group 3 on days 14 and 17 (*p* = 0.017, *p* = 0.018, respectively), but no statistical significance has been observed on days 20, 23, 26, and 28.

While the number of scratching movements of the animals in the fourth group given fish oil was significantly higher than on the 14th and 17th days compared to the first day (*p* = 0.018), no statistical significance was observed on days 20, 23, 26, and 28 (*p* = 0.323) (Table 3).

**Table 3:** Average nose scratching movements by groups.

Groups	Day 1	Day 14	Day 17	Day 20	Day 23	Day 26	Day 28
Group 1 Control Group	1.28	1.43	2.15	1.44	1.87	1.87	1.87
Group 2 Allergic Rhinitis Control Group	1.00	8.43^a^	14.41^a^	18.56^a^	21.9^a^	22.68^a^	23.35^a^
Group 3 AR Group given montelukast treatment	1.14	9.13^a^	4.38^a^	1.58^b^	1.58^b^	1.85^b^	1.41^b^
Group 4 AR Group given omega-3 fatty acid oil	1.16	9.16^a^	4.41^a^	1.63^b^	1.64^b^	1.88^b^	1.45^b^

AR, Allergic Rhinitis.

a *p* <  0.05.

b *p* >  0.05.

There was no statistical difference in the number of sneezing days compared to montelukast and omega-3 fatty acids groups.

## Histological evaluation

No eosinophil infiltration was detected in the control and montelukast groups, while 42.9% of rats in the allergic rhinitis group had mild, and 57.1% had moderate eosinophil infiltration. Mild eosinophil infiltration was detected in 14.3% of rats in the omega-3 group. The difference between the groups in terms of eosinophil infiltration was statistically significant (*p* < 0.001) (Table 4).

**Table 4:** Comparison of eosinophil infiltration by groups.

			Groups				Total	χ^2^	*p-*value
			Control Group	Allergic Rhinitis Group	Montelukast Treatment Group	Omega-3 Treatment Group			
Eosinophil Infiltration	None	n	7	0	7	6	20	24.800	0.000
		%	100	0.0	100	85.7	71.4		
	Mild	n	0	3	0	1	4		
		%	0.0	42.9	0.0	14.3	14.3		
	Moderate	n	0	4	0	0	4		
		%	0.0	57.1	0.0	0.0	14.3		
Total		n	7	7	7	7	28		
		%	100	100	100	100.0	100.0		

None of the rats in the control, montelukast, and omega-3 groups had cilia loss. On the contrary, 28.6% of mice in the allergic rhinitis group had mild loss, and 71.4% had moderate cilia loss, and the difference between the groups was statistically significant (*p* < 0.001) (Table 5).

**Table 5:** Comparison of cilia loss by groups.

			Groups				Total	χ^2^	*p-*value
			Control	Allergic Rhinitis Group	Control	Allergic Rhinitis Group			
Cili Loss	None	n	7	0	7	7	21	28.000	0.000
		%	100	0.0	100	100	75.0		
	Mild	n	0	2	0	0	2		
		%	0.0	28.6	0.0	0.0	7.1		
	Moderate	n	0	5	0	0	5		
		%	0.0	71.4	0.0	0.0	17.9		
Total		n	7	7	7	7	28		
		%	100	100	100	100	100.0		

None of the rats in the montelukast and omega-3 groups had any increase in goblet cells, whereas 14.3% of the rats in the control group and 28.6% in the allergic rhinitis group had a mild increase. Last but not least, 71.4% of rats in the allergic rhinitis group had a moderate increase. The difference between the groups was statistically significant (*p* < 0.001) (Table 6).

**Table 6:** Comparison of goblet cell growth by groups.

			Groups				Total	χ^2^	*p-*value
			Control Group	Allergic Rhinitis Group	Montelukast Treatment Group	Omega-3 Treatment Group			
Goblet Cell Growth	None	n	6	0	7	7	20	25.467	0.000
		%	85.7	0.0	100	100	71.4		
	Mild	n	1	2	0	0	3		
		%	14.3	28.6	0.0	0.0	10.7		
	Moderate	n	0	5	0	0	5		
		%	0.0	71.4	0.0	0.0	17.9		
Total		n	7	7	7	7	28		
		%	100	100	100	100	100		

Mild vascular proliferation was observed in 28.6% in the control group, 71.4% in the allergic rhinitis group, and 28.6% in the omega-3 group. Moderate vascular proliferation has been detected in 28.6% of the montelukast group and 42.9% of the omega-3 group (*p* < 0.05) (Table 7).

**Table 7:** Comparison of vascular proliferation by groups.

			Groups				Total	χ^2^	*p-*value
			Control Group	Allergic Rhinitis Group	Montelukast Treatment Group	Omega-3 Treatment Group			
Vascular Proliferation	None	n	5	0	5	4	14	12.857	0.045
		%	71.4	0.0	71.4	57.1	50		
	Mild	n	2	5	2	3	12		
		%	28.6	71.4	28.6	42.9	42.9		
	Moderate	n	0	2	0	0	2		
		%	0.0	28.6	0.0	0.0	7.1		
Total		n	7	7	7	7	28		
		%	100	100	100	100	100		

Chondrocyte hypertrophy was not observed in any of the rats in the control group. On the other hand, mild chondrocyte hypertrophy was denoted in 71.4% in the allergic rhinitis group, 14.3% in the montelukast group, and 28.6% in the omega-3 group (*p* < 0.05) (Table 8).

**Table 8:** Comparison of hypertrophy level in chondrocytes according to groups.

			Groups				Total	χ^2^	*p-*value
			Control Group	Allergic Rhinitis Group	Montelukast Treatment Group	Omega-3 Treatment Group			
Chondrocyte Hypertrophy	None	n	7	0	6	5	18	19.444	0.003
		%	100	0.0	85.7	71.4	64.3		
	Mild	n	0	5	1	2	8		
		%	0.0	71.4	14.3	28.6	28.6		
	Moderate	n	0	2	0	0	2		
		%	0.0	28.6	0.0	0.0	7.1		
Total		n	7	7	7	7	28		
		%	100	100	100	100	100		

**Fig. 1:**
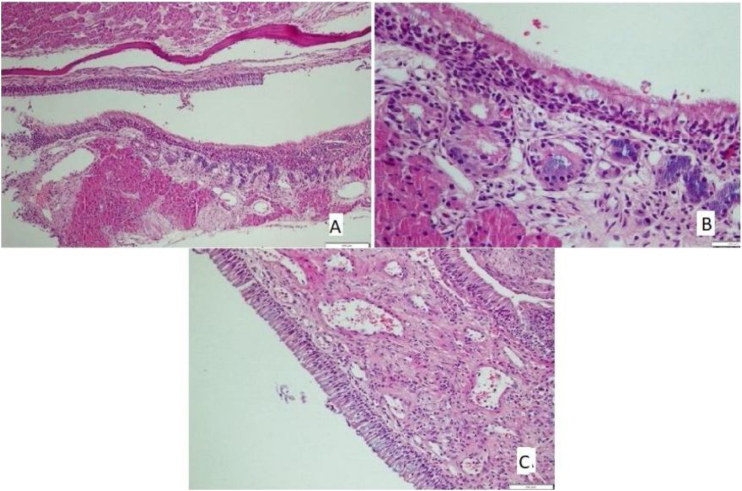
(A) Goblet cell hyperplasia, increased inflammation, loss of cilia, and increased eosinophils in Allergic Rhinit model (HE × 40). (B) Congestion and increased vascularisation in Allergic Rhinit model (HE × 40). (C) Chondrosithipertrophy in Allergic Rhinit model (HE × 40).

**Fig. 2:**
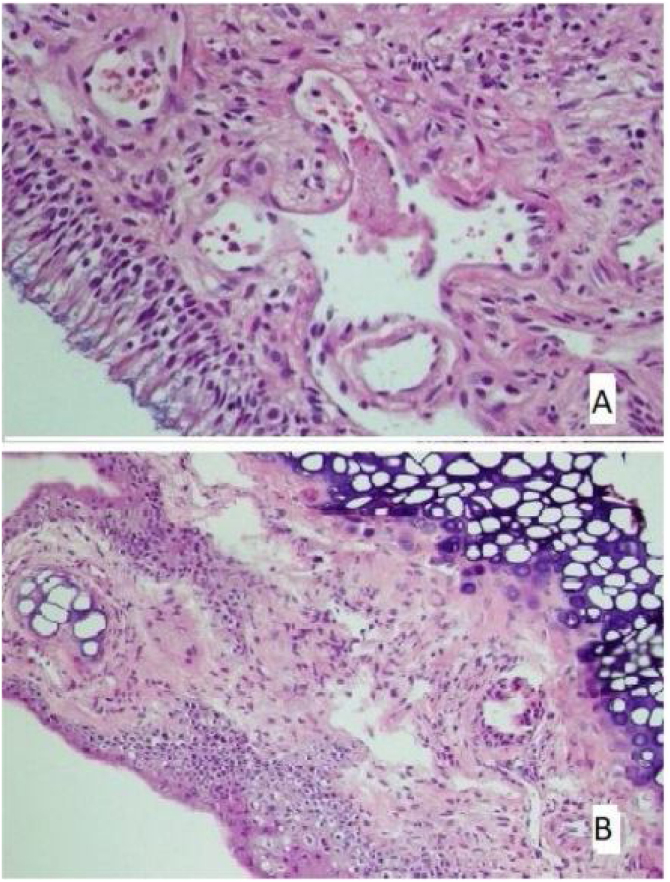
(A) The section in which the cilia are clearly observed, and there is no inflammation and eosinophil increase in the montelukast treatment group (HE × 40) and (B) the section where inflammation is not observed, cilia are preserved, and goblet cell hyperplasia is not observed in the omega-3 fatty acid oil treatment group (HE × 40).

The histological differences between the groups can be seen in Fig. 1, Fig. 2.

## Discussion

Allergic rhinitis is an inflammation and a multi-factor inflammatory disease characterized by heterogeneous clinical features, histopathology, and therapeutic response.^7^, ^8^ In some studies, the pathogenesis and pathophysiology of chronic rhinitis have been investigated using animal models such as rabbits and mice.^9^, ^10^, ^11^ To mimic allergic or eosinophilic rhinitis, mice are sensitized using OVA intraperitoneally to the sinonasal space.^12^ However, inhalation of OVA alone in asthma mice does not elicit a broad spectrum of allergic change or overcome airway tolerance if given without an adjuvant.^13^, ^14^ Also, OVA alone does not cause chronic rhinitis in humans. Inhalation of Alternaria, Cladosporium, and Aspergillus fungi plays a role in human allergic airway diseases such as asthma and allergic rhinitis.^15^

In particular, activation of the Protease-Activated Receptor (PAR) in fungal protection is responsible for the allergic response in the upper respiratory tract, as in rhinitis and Nasal Polyps (NPs).^16^ Fungal proteases may be more specific triggers of allergic rhinitis. In an asthmatic mouse model, histological and immunological features with eosinophilic infiltration.^17^, ^18^ This model increased OVA-associated fungal proteases, eosinophils, and Th_2_ cells, causing allergic inflammation.^14^ Epidemiological studies suggest that the increase in the prevalence of allergic diseases in Western societies is related to diet. The major reason is the reduced consumption of long-chain Omega-3 fatty acids. It is observed that atopy develops less frequently in societies that are fed a diet rich in omega-3 fatty acids.^19^

Omega-3 fatty acids are necessary for the body and are taken as external supplements because they cannot be produced in the human body. Oil with significant omega-3 acids includes α-linolenic acid, Eicosapentaenoic Acid (EPA), and Docosahexaenoic Acid (DHA). The term omega-3 (“n-3” also used as “w-3”) means that the first double bond is the third carbon-carbon bond when counted from the methyl group at the end of the carbon chain (w). These three unsaturated oils have 3, 5, or 6 double bonds in a chain of 18, 20, or 22 carbons. Epidemiological data show that dietary factors may play a role in the recent increase in allergic disease prevalence. Omega-3 fatty acids are thought to have primarily anti-inflammatory effects and play a role in immune responses such as allergies.^20^

Many studies have been carried out in perinatal life to prevent allergic diseases with the support of w-3 fatty acid. In a randomized controlled study of 98 atopic women, fish oil and a placebo were given to two groups from 20-weeks of gestation to delivery. When babies in the fish oil group reached age 1, the prevalence of developing sensitization against eggs was lower. Although the prevalence of atopic disease did not decrease, these children had milder dermatitis than the control group. There was no difference between IgE levels in the study groups.^21^ The main symptoms of allergic rhinitis in humans are sneezing, an itchy nose, and a runny nose. Therefore, animal models with similar allergic symptoms were needed to evaluate the effectiveness of antiallergic drugs. In this study, it was observed that typical allergic rhinitis symptoms such as sneezing and nasal scratching in rats were observed with repeated topical intranasal OVA-protease applications. It has been demonstrated that montelukast and omega-3 given for therapeutic purposes significantly reduce sneezing and nasal scratching due to antigen-antibody interaction.

Significant results have been obtained in our study, similar to the literature. As a result, the number of sneezing and nasal scratching movements decreased significantly in the group given montelukast and fish oil treatment.

A significant reduction in typical allergic rhinitis symptoms, such as a scratching nose and sneezing, has been reported in animals. Similarly, the study conducted by Avinçsal^22^ reported that the number of sneezing and nasal scratching movements in mice receiving doxycycline treatment decreased significantly. Dunstan et al.^23^ showed that fish oil supplementation during pregnancy increased the rate of DHA in newborn cell membranes, while w-6 fatty acid decreased arachidonic acid levels. Changing the contents of the cell membranes can change the immune response. Treatment of w-3 fatty acid during pregnancy affects cytokine levels in newborn cord blood cells. It has been shown that IL-13 levels in the cord serum of children given prenatal fish oil decreased significantly compared to those given the placebo. In addition, Dunstan et al.^23^ stated that some cytokine levels decreased, and lymphoproliferative immune response was inhibited in children who encountered w-3 fatty acids before birth. As in human studies, animal experiments elaborated that w-3 fatty acid support affects neonatal immune functions early in life; thus, providing w-3 fatty acid support in the early stages of life is a promising approach to preventing allergic diseases.

Cardoso et al.^24^ also created a food allergy mouse model to demonstrate food-induced intestinal inflammation. They sensitized mice with peanut seeds and evaluated eosinophils in the mouse gut's Hematoxylin Eosin (HE) dye and mast cells in toluidine dye. As a result, they found significantly increased eosinophil and mast cell counts in the intestinal mucosa.

Eosinophil accumulation in tissues is a prominent feature of the allergic response. The direct relationship between the severity of food allergy and the activation of gastrointestinal eosinophils has been well established. In addition, eosinophils act as APC (antigen-presenting cells) in inflamed tissue and increase the Th2 response. In healthy cases, these cells are found in the lamina propria of the gastric and intestinal mucosa. However, in patients with food allergies, the distribution of eosinophils (intraepithelial, lamina propria, submucosa), shape, and function differ because these cells are easily activated through IgE receptors. Thus, it can be said that eosinophils contribute to the initiation of the type I reaction in the skin and mucosa after exposure to the allergen.^25^

Eotaxin and IL-5 are major chemokines responsible for eosinophil activation. IL-5 plays an essential role in the proliferation and differentiation of eosinophils in the bone marrow and regulates its function in inflamed tissue. Eotaxin is also responsible for the accumulation of eosinophils in the allergic zone. Local and peripheral eosinophilia is observed when mice are orally provoked with food allergens.^25^ In this mouse model investigating the effect of omega-3 fatty acids on food allergy, it was thought that it would be beneficial to evaluate the eosinophil counts in the mouse intestinal mucosa.

Many animal models have demonstrated the clinical, biochemical, and immunological benefits of dietary fatty acids. These include increased life expectancy, decreased proteinuria and anti-DNA antibodies in glomerulonephritis, decreased joint inflammation in collagen-induced arthritis, and decreased inflammation in colitis.^26^ These observations showed that montelukast and w-3 fatty acids can also be helpful in these diseases in humans.

The metabolism and generation of bioactive lipid mediators are key events in exerting the beneficial effects of dietary omega-3 fatty acids in regulating allergic inflammation. This is quite similar to that of montelukast, which causes inhibition of airway cysteinyl leukotriene receptors. In an early cross-sectional human study by Hoff et al., n-3 fatty acids in red blood cell membranes (EPA) or diet (ALA) were associated with a decreased risk of allergic sensitization and allergic rhinitis. A higher dietary intake of w-3 was associated with a decreased risk of allergic sensitization and allergic rhinitis.^27^ Sawane et al. conducted an animal study and found that dietary linseed oil, which contains high amounts of Alpha-Linolenic Acid (ALA), dampened allergic rhinitis through eosinophilic production of 15-Hydroxy Eicosapentaenoic acid (15-HEPE), a metabolite of Eicosapentaenoic Acid (EPA). Lipidomic analysis revealed that 15-HEPE was particularly accumulated in the nasal passage of linseed oil-fed mice after the development of allergic rhinitis with the increasing number of eosinophils. Indeed, the conversion of EPA to 15-HEPE was mediated by the 15-lipoxygenase activity of eosinophils. Intranasal injection of 15-HEPE dampened allergic symptoms by inhibiting mast cell degranulation, which was mediated by the action of peroxisome proliferator-activated receptor gamma. These findings identify 15-HEPE as a novel EPA-derived and eosinophil-dependent anti-allergic metabolite and provide a preventive and therapeutic strategy against allergic rhinitis.^28^

Dietary modification can influence the severity of asthma and reduce the prevalence and incidence of this condition. A possible contributing factor to the increased incidence of asthma in Western societies may be consuming a pro-inflammatory diet. In the typical Western diet, 20‒25-fold more omega (n)-6 Polyunsaturated Fatty Acids (PUFA) than n-3 PUFA are consumed, releasing pro-inflammatory arachidonic acid metabolites. Eicosapentaenoic acid and docosahexaenoic acid are n-3 PUFA derived from fish oil that competitively inhibits n-6 PUFA Arachidonic Acid (AA) metabolism, and this reduces the generation of pro-inflammatory 4-series Leukotrienes (LTs) and 2-series Prostaglandins (PGs) and production of cytokines from inflammatory cells. These data are consistent with the proposed pathway by which dietary intake of n-3 PUFA modulates pulmonary inflammation and reactivity.^29^ Omega-3 – 3 Fatty acids are key in signaling and producing mediators in the allergic and inflammatory pathways. A study of 38 grass pollen allergic asthmatics amelioration in bronchial allergic inflammation has been observed,^30^ consistent with our findings. As seen in the aforementioned research, both montelukast and omega-3 have substantial effects on reducing inflammation.^4^, ^27^, ^28^, ^29^, ^30^

## Conclusion

Regarding the results of this research, it was observed that montelukast and omega-3 fatty acids had histopathological and clinical antiallergic effects in the allergic rhinitis model. We believe that further randomized controlled trials incorporating larger cohorts are warranted to verify the use of omega-3 fatty acids in treating allergic rhinitis.

## Statements and declarations

The authors have no competing interests to declare that are relevant to the content of this article. No funds, grants, or other support was received.

## Ethical declaration

Ethics committee approval has been granted from our institution (2016/145).

## Funding

There is no specific funding related to this research.

## Conflicts of interest

The authors declare no conflicts of interest.
